# Aberrant B cell receptor signaling responses in circulating double-negative 2 B cells from radiographic axial spondyloarthritis patients

**DOI:** 10.1016/j.jtauto.2025.100270

**Published:** 2025-01-23

**Authors:** Rick Wilbrink, Stefan F.H. Neys, Rudi W. Hendriks, Anneke Spoorenberg, Frans G.M. Kroese, Odilia B.J. Corneth, Gwenny M.P.J. Verstappen

**Affiliations:** a Department of Rheumatology and Clinical Immunology, University of Groningen, University Medical Center Groningen, Groningen, The Netherlands; b Department of Pulmonary Medicine, Erasmus MC, University Medical Center, Rotterdam, The Netherlands

**Keywords:** Axial spondyloarthritis, Radiographic axial spondyloarthritis, Ankylosing spondylitis, B cells, B cell receptor (BCR) signaling, Phosphoflow cytometry

## Abstract

**Objective:**

Radiographic axial spondyloarthritis (r-axSpA) is a chronic rheumatic disease in which innate immune cells and T cells are thought to play a major role. However, recent studies also hint at B cell involvement. Here, we performed an in-depth analysis on alterations within the B-cell compartment from r-axSpA patients.

**Methods:**

We performed immune gene expression profiling on total peripheral blood B cells from 8 r-axSpA patients and 8 healthy controls (HCs). Next, we explored B cell subset distribution and B-cell receptor (BCR) signaling responses in circulating B cells from 28 r-axSpA patients and 15 HCs, by measuring spleen tyrosine kinase, phosphoinositide 3-kinase and extracellular signal regulated kinase 1/2 phosphorylation upon α-Ig stimulation using phosphoflow cytometry.

**Results:**

Immune gene expression profiling indicated an elevated pathway score for BCR signaling in total B cells from r-axSpA patients compared with HCs. Flow cytometric analysis revealed an increase in frequency of both total and double-negative 2 (DN2) B cells in r-axSpA patients compared with HCs. In r-axSpA patients, DN2 B cells displayed an isotype shift towards IgA. Remarkably, where DN2 B cells from HCs were hyporesponsive, these cells displayed significant proximal BCR signaling responses in r-axSpA patients. Enhanced BCR signaling responses were also observed in the transitional and naïve B cell population from r-axSpA patients compared with HCs. The enhanced BCR signaling responses in DN2 B cells correlated with clinical disease parameters.

**Conclusion:**

In r-axSpA patients, circulating DN2 B cells are expanded and, together with transitional and naïve B cells, display significantly enhanced BCR signaling responses upon stimulation. Together, our data suggest B cell involvement in the pathogenesis of r-axSpA.

## Graphical abstract

**Figure:**
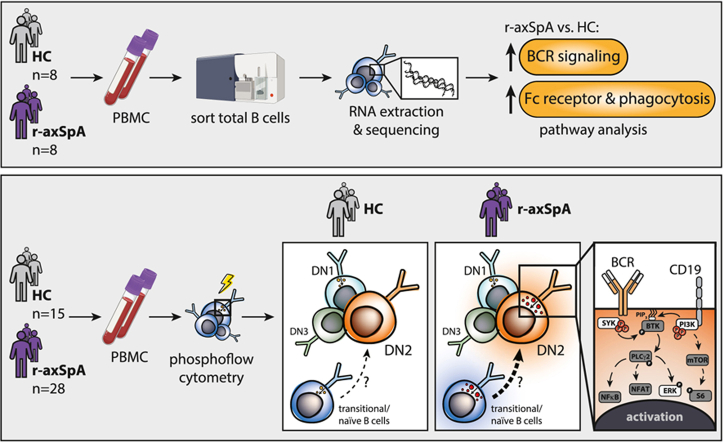

## 1 Introduction

Radiographic axial spondyloarthritis (r-axSpA), formerly known as ankylosing spondylitis, is a chronic, rheumatic disease characterized by inflammation of the axial skeleton, leading to aberrant bone remodeling [1]. A substantial proportion of r-axSpA patients will also develop extra-skeletal manifestations (ESM) including psoriasis, inflammatory bowel disease, and uveitis [2]. Current evidence suggests that the underlying pathophysiology of r-axSpA is multifaceted, involving innate immune cells, such as neutrophils and macrophages, as well as T cells [3]. According to current dogma, the role of B cells in r-axSpA pathogenesis is thought to be marginal. This assumption largely stems from the absence of disease-defining autoantibodies [3]. Nevertheless, recent studies indicate that the role of B cells in the pathogenesis of r-axSpA may have been underestimated. This is exemplified by B cell infiltration in r-axSpA-affected tissues and disturbances in the composition of the peripheral blood B cell compartment of r-axSpA patients [4]. Furthermore, treatment with rituximab, a B-cell depleting therapy, resulted in clinical improvement in a subgroup of r-axSpA patients, in particular patients who were treatment-naïve for anti-TNFα [5,6].

Previously, we observed elevated frequencies of CD27^−^CD21^lo^ B cells in blood from r-axSpA patients [7]. CD21^lo^ B cells represent a heterogeneous population of cells defined by a low expression or lack of complement receptor 2 (CD21). During chronic inflammatory conditions, such as persistent viral infections, but also in autoimmune diseases including Sjögren's disease (SjD), systemic lupus erythematosus (SLE), and rheumatoid arthritis (RA), this population is expanded in the circulation [8,9]. Further subdivision of CD21^lo^ B cells based on the expression of CD11c (CD21^lo^CD11c^+^ B cells) characterizes a population of B cells thought to have been (recently) activated [10,11]. CD21 and CD11c are also used for further subdivision of the double negative (DN; CD27^–^IgD^–^) B cell population into DN1 (CD21^+^CD11c^–^), DN2 (CD21^lo^CD11c^+^), and DN3 (CD21^lo^CD11c^–^) cells [12]. DN2 B cells in particular are thought to play a role in the pathogenesis of systemic autoimmune diseases, such as SLE, where DN2 B cells are associated with an extra-follicular origin and poised differentiation towards antibody secreting cells (ASC) [13]. Interestingly, the CD27^−^CD21^lo^ B cell population, which contains DN2 B cells, may comprise a substantial proportion of cells expressing autoreactive B cell receptors (BCR) [14,15]. In addition, CD27^−^CD21^lo^ B cells have been put forward as a reservoir of anergic cells, exemplified by a lack of calcium efflux and diminished upregulation of B cell activation markers and proliferation upon BCR stimulation [[14], [15], [16]].

BCR signaling plays a pivotal role in B cell development, activation, proliferation, and differentiation. Upon BCR stimulation, multiple downstream signaling pathways are activated. These events involve the phosphorylation of several protein kinases, including spleen tyrosine kinase (SYK), phosphoinositide 3-kinase (PI3K), Bruton's tyrosine kinase (BTK), and extracellular signal regulated kinase (ERK)1/2 [17]. Enhanced BCR signaling may play an important role in the pathogenesis of human autoimmune disease, such as RA and granulomatosis with polyangiitis (GPA) [[18], [19], [20], [21]].

Because of multiple similarities in subset distribution of circulating B cells between r-axSpA and rheumatic autoimmune disease patients [4], we sought to investigate BCR signaling responses in the peripheral B cell compartment of r-axSpA patients. To this end, in the work described here, we performed immune gene expression profiling on circulating total B cells obtained from r-axSpA patients and healthy controls (HCs) to explore potential differences in genes and pathways related to B cell activation. Subsequently, in a larger cohort of r-axSpA patients and HCs, BCR signaling responses were assessed in various B cell subpopulations using phosphoflow cytometry. Given our recent finding of expanded CD27^−^CD21^lo^ B cells in r-axSpA patients and the link of these cells with autoimmune disease [7,14,15], special emphasis was given to the DN B cell compartment. Finally, BCR signaling responses in r-axSpA patients were correlated with clinical disease parameters.

## 2 Material and methods

### 2.1 Patient and control samples

Whole blood was obtained from a total of 36 r-axSpA patients, participating in the Groningen-Leeuwarden axSpA (GLAS) cohort, who visited the outpatient clinic of the department of Rheumatology and Clinical Immunology at the University Medical Center Groningen, The Netherlands [22]. These r-axSpA patients were diagnosed with r-axSpA by their rheumatologist and fulfilled the Assessment in Spondyloarthritis international Society (ASAS) classification criteria for r-axSpA [23]. Included r-axSpA patients did not receive any biologic (e.g., anti-TNFα), synthetic (e.g., JAK/STAT-inhibitors) nor conventional disease-modifying anti-rheumatic drugs within six months prior to blood donation. Via Sanquin Blood Supply Foundation, The Netherlands, buffy coats were obtained from 8 HCs and whole blood from 15 HCs. HCs were sex- and age-matched to the patients. Peripheral blood mononuclear cells (PBMCs) were isolated from blood samples and cryopreserved at −150 °C until further use. The medical research ethics committee of the Medical Center Leeuwarden provided approval for this study (RTPO 364/604) and all patients and HCs provided written informed consent, in accordance with the Declaration of Helsinki. Patients and controls were split in two independent experimental cohorts. Immune gene profiling was conducted in a cohort of 8 r-axSpA patients and 8 HCs, and phosphoflow cytometry was performed in another cohort of 28 r-axSpA patients and 15 HCs. Patient and HC characteristics of both experimental settings are described in Suppl. Table 1.

### 2.2 B cell sorting and RNA isolation

Cryopreserved PBMCs were thawed and washed in RPMI +10 % fetal bovine serum (FBS) at 37 °C. Subsequently, cells were resuspended in phosphate buffer saline (PBS; ThermoFisher, Massachusetts, United States of America), supplemented with 0.5 % bovine serum albumin (BSA, ThermoFisher) and 2 mM ethylenediaminetetraacetic acid (ThermoFisher). The EasySep™ Human CD3 Positive Selection Kit (STEMCELL Technologies, Cologne, Germany) was used to deplete T cells to minimize cell sorting time. CD19^+^CD20^+^ B cells were subsequently sorted with the MoFlo Astrios (Beckman Coulter) using the gating strategy shown in Suppl. Fig. 1. B cells were captured in safe-lock Eppendorf tubes (Qiagen, Hilden, Germany) containing 350 μL RNeasy Lysis Buffer (RLT, Qiagen) and β-mercaptoethanol (10 μL/mL) was added to denature RNAses. Subsequently, RNA was isolated using the RNeasy Micro Kit (Qiagen) and vials were stored at −80 °C until further use.

### 2.3 Immune gene profiling

The NanoString Technologies (Seattle, WA, USA) nCounter® Autoimmune Profiling Panel was utilized to quantify the expression of genes per manufacturer's instructions. RNA integrity was measured by the Agilent TapeStation (Santa Clara, CA, USA) and concentration was measured with Qubit (ThermoFisher). Sample input was 100 ng RNA (20 ng/μl). Hybridizations were performed at 67 °C for 16 h (o/n) and RNA/probe complexes (Autoimmune Profiling codeset, Nanostring Technologies) were loaded on the cartridge. Samples were randomly divided in batches with four subjects per group over two cartridges and run separately on a nCoulter SPRINT platform (Nanostring Technologies, Seattle, WA). Data analysis was conducted through NanoString's nSolver Advanced Analysis Software (version 2.0.134). Genes with a minimum of 20 read counts across samples were included in the analysis. Pathway scores were calculated as the first principal component (1st eigenvectors) of the normalized expression of related pathway genes. No significant outliers were observed among the samples.

### 2.4 B cell receptor stimulation *in vitro*

BCR signaling responses were assessed on a functional level by phosphoflow cytometry, as previously described [24]. In short, frozen PBMCs were thawed and resuspended in ice-cold RPMI containing 5 % FBS. Fractions of 1.0 × 10^6^ cells per well were plated in a 96-well plate, brought to 37 °C, and incubated with fixable live/dead marker BV575 (BD Biosciences) for 10 min. During the last 5 min, cells were either stimulated with BCR-stimulating goat-anti-human immunoglobulin (α-Ig) F(ab)_2_ fragments (20 μg/mL; SouthernBiotech) or were left unstimulated. The reaction was stopped by fixation with eBioscience FoxP3/Transcription Factor Staining Fixative (ThermoFisher Scientific). Subsequently, cells were stained intracellularly for markers to distinguish B cell subsets and with antibodies recognizing phosphorylated protein to quantify BCR signaling. For the latter, three targets were analyzed based on i) identical kinases and phosphorylation sites studied previously in systemic autoimmune disease patients, ii) involvement in different pathways downstream of the BCR, and iii) showing the highest stimulation index. These included phosphorylated (p)SYK (Y348), pPI3K p85 (Y458), and pERK1/2 (T202/Y204). Antibodies are listed in Suppl. Table 2. Samples were measured using a FACSymphony A5 flow cytometer (BD Biosciences).

Flow cytometry data were analyzed with FlowJo Version 10.9.0. (BD Biosciences). Quality control was performed on raw data using the Peak Extraction and Cleaning Oriented Quality Control (PeacoQC) plug-in [25]. Because of compromised IgD expression after α-Ig stimulation, double negative (DN) B cells were gated as being CD19^+^CD38^lo^CD27^–^IgM^–^ instead of CD19^+^CD38^lo^CD27^–^IgD^–^. Unstimulated samples showed that these two populations were ∼99 % overlapping. After gating for different B cell subpopulations, the geometric mean fluorescence intensity (gMFI) was used to quantify the phosphorylation signal. The response to stimulation was depicted as the stimulation index, which was calculated by dividing the gMFI values following stimulation by the gMFI in unstimulated conditions.

### 2.5 Statistical analysis

To test for parametric distribution of data with each comparison, histograms or QQ-plots were analyzed. An unpaired *t*-test was used in the case of parametric distribution of the data, whereas for a non-parametric distribution, a Mann–Whitney *U* was conducted. When comparing more than two groups, a one-way ANOVA with Tukey's multiple comparisons test (parametric) or a Kruskal-Wallis with Dunn's test to correct for multiple comparisons (non-parametric) was used. Spearman's rank correlation coefficients were calculated for correlation analyses. A correlation coefficient of <0.40 was interpreted as weak, 0.40–0.59 as moderate, 0.60–0.80 as strong, and >0.80 as excellent. P-values <0.05 were considered statistically significant, and any P-value corrections are explicitly detailed in the figure captions. Statistical testing and visualization were carried out using GraphPad Prism 9.5.1 software (GraphPad Prism Inc., San Diego, CA, USA) or R version 4.1.1.

## 3 Results

### 3.1 Altered expression of signature genes of B cell receptor signaling in B cells from r-axSpA patients

**Fig. 1:**
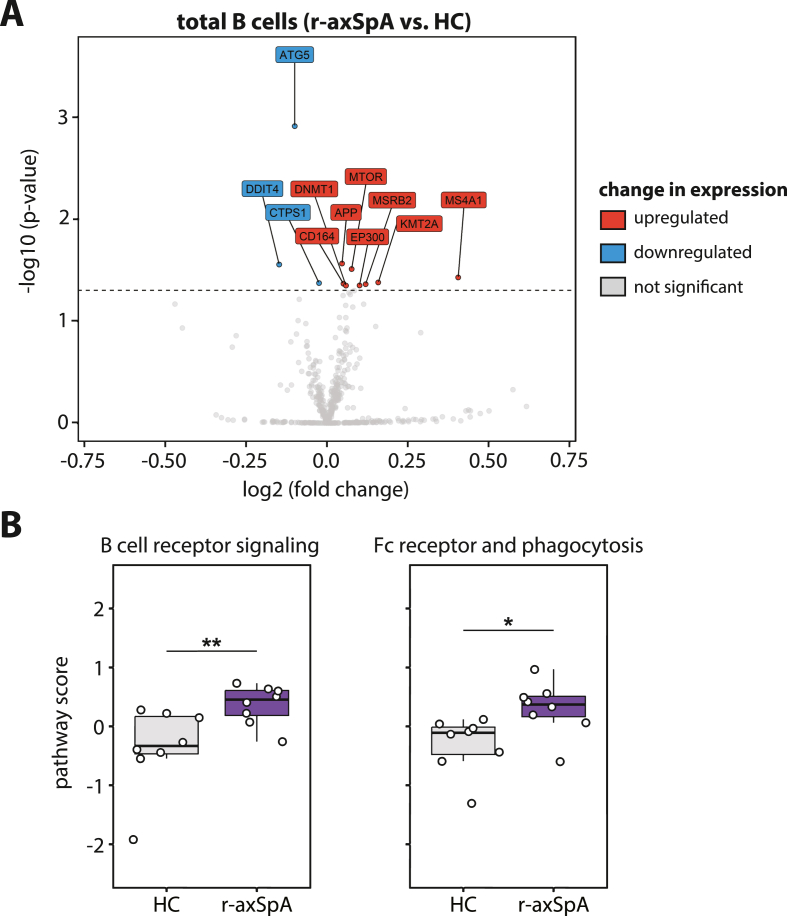
**Elevated pathway scores indicative for overall upregulated expression of genes involved in B cell receptor signaling in B cells from r-axSpA patients.** (**A**) Volcano plot demonstrating differentially expressed genes in total B cells from radiographic axial spondyloarthritis (r-axSpA) patients compared with healthy controls (HC; both N = 8). (**B**) Pathway scores from ‘BCR signaling’ and ‘Fc receptor and phagocytosis’ of total B cells from r-axSpA patients in comparison with those from HCs (both N = 8). The horizontal line in the boxplot represents the median with jitter points for individual data points. Differences between HCs and r-axSpA patients were analyzed by a Mann-Whitney *U*. ∗*P* < 0.05; ∗∗*P* < 0.01.

To identify possible alterations in pathways related to B cell activation in r-axSpA patients, we sorted circulating B cells from eight r-axSpA patients and eight HCs and performed bulk RNA expression assays (gating strategy shown in Suppl. Fig. 1). Differential gene expression analysis revealed eight significantly upregulated and three significantly downregulated genes between r-axSpA patients and HCs (Fig. 1A). Genes upregulated in B cells from r-axSpA patients included *MTOR* and *MS4A1*. *MTOR* encodes a protein kinase that is involved in many different signaling pathways and regulates fundamental cellular processes, from protein synthesis to autophagy [26]. The *MS4A1* gene encodes the surface protein CD20, known as a B-cell lineage marker and thought to play a role in activation of B cells [27]. One of the downregulated genes encodes for the ATG5 protein. Besides being required for autophagy, ATG5 participates in BCR clustering and polarization and is involved in antigen presentation [28]. Although we observed differentially expressed genes in B cells from r-axSpA patients, none passed the threshold for significance after p-value adjustment (Holm-Bonferroni).

Next, pathway analysis was performed to identify changes in biological processes involved in the activation of B cells from r-axSpA patients. Pathway scores were generated for multiple pathways comprising a related set of genes, with positive scores indicating mostly upregulated genes, and negative scores containing more downregulated genes. Two pathways related to B cell activation showed significantly higher scores in B cells from r-axSpA patients compared with HCs: 1) BCR signaling and 2) Fc receptor and phagocytosis (Fig. 1B, Suppl. Fig. 2). Of note, these pathways share 65% of the genes, such as *SYK,* *LYN*, *BTK*, and *PIK3CA*, which may partly explain the similar directionality of the pathway scores. In addition to pathways related to B cell activation, the autoantigens-pathway score was significantly higher in r-axSpA patients compared with HCs (see Suppl. Fig. 2). A list of the genes that comprise the pathways with a significantly different pathway score are listed in Suppl. Table 3. While scores for other pathways hinted at down-regulation in r-axSpA patients, none reached statistical significance (Suppl. Fig. 2).

In summary, our results revealed altered expression of BCR signaling, Fc receptor and phagocytosis, and autoantigen-associated gene signatures, suggesting a possible dysregulation in B cell signaling pathways in circulating B cells from r-axSpA patients.

### 3.2 DN2 B cells are expanded in the circulation of r-axSpA patients

**Fig. 2:**
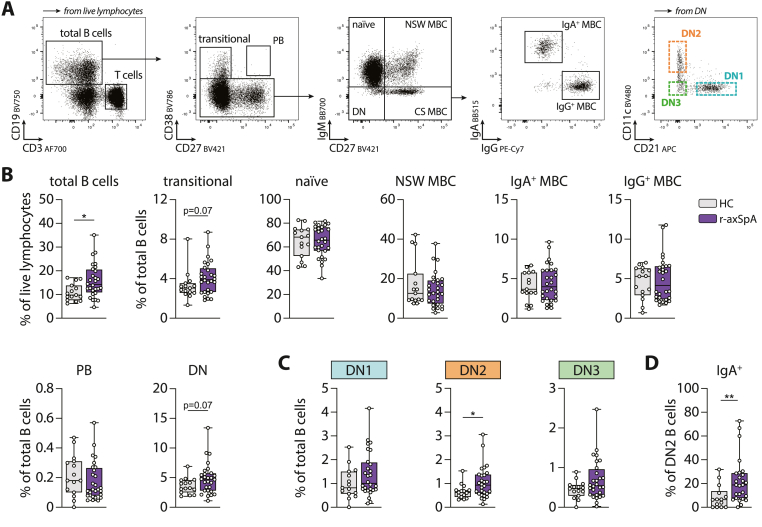
**Altered B cell subset and isotype distribution in r-axSpA patients.** (**A**) Gating strategy for the detection of different circulating B cell subsets, including DN1 (CD21^+^CD11c^−^; blue), DN2 (CD21^lo^CD11c^+^; orange), and DN3 (CD21^lo^CD11c^−^; green) B cells. (**B**) The percentage of total B cells from live lymphocytes, and the distribution of the indicated B cell subsets within the total B cell population in HCs (N = 15) and r-axSpA patients (N = 28). (**C**) The percentage of DN1, DN2, and DN3 B cells from the total B cell population. (**D**) The percentage of IgA ^+^ cells within the DN2 B cell population. The horizontal line in the boxplots represent the median with jitter points for individual data points. Differences were analyzed by an unpaired *t*-test or a Mann-Whitney *U*. ∗*P* < 0.05; ∗∗*P* < 0.01. PB, plasmablasts; MBC, memory B cells; CS, class-switched; NSW, non-switched; DN, double negative.

As a next step, we studied the composition of circulating B cell subsets in r-axSpA patients (n = 28) compared with HCs (n = 15; see Fig. 2A for gating strategy). In r-axSpA patients, the percentage of total B cells within the live lymphocyte population was increased (Fig. 2B). Apart from a trend towards elevated percentages of transitional and double negative (DN) B cells, no significant alterations were detected in percentages of naïve B cells, IgM^+^, IgA^+^, and IgG^+^ memory B cells (MBC), or plasma blasts (PB) in r-axSpA patients (Fig. 2B).

As a next step, the different DN B cell subpopulations were investigated. To ensure accurate qualitative measurements, the number of cells analyzed within each population were equal between patients and HCs, and an average of ≥100 cells were included within each DN B cell gate (data not shown). Here, an increase in the frequency of DN2 B cells within the total B cell compartment was found in r-axSpA patients, whereas no significant changes were seen for DN1 and DN3 B cells (Fig. 2C). Correspondingly, DN2 B cells were significantly enriched within the total DN B cell population (data not shown). Within the DN2 B cell population, r-axSpA patients displayed increased percentages of IgA^+^ cells and decreased percentages of IgG^+^ cells (Fig. 2D, and data not shown).

Together, r-axSpA patients showed increased percentages of DN2 B cells in the circulation compared with HCs, which was accompanied by a shift in isotype towards IgA.

### 3.3 DN2 and DN3 B cells are anergic in healthy controls

**Fig. 3:**
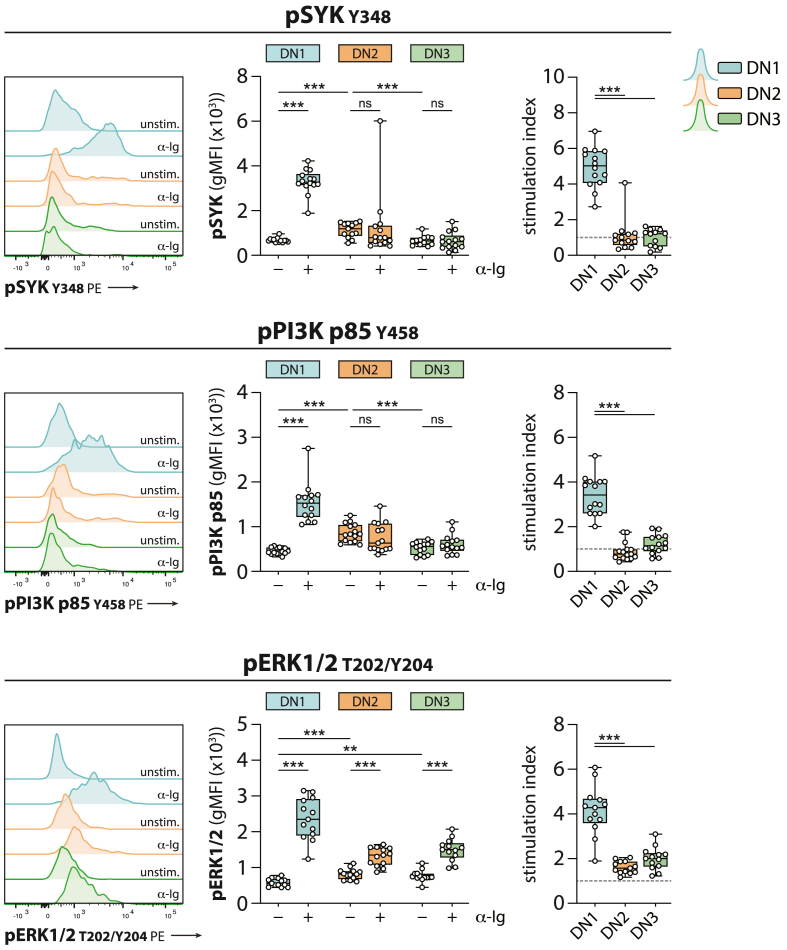
**DN2 and DN3 B cells are hyporesponsive to BCR stimulation in homeostatic conditions.** The phosphorylation of SYK (Y348), PI3K p85 (Y458), and ERK1/2 (T202/Y204) was measured under unstimulated conditions (– α-Ig) and following BCR stimulation (+α-Ig) in DN1 (blue), DN2 (orange), and DN3 (green) B cells from HCs. Representative histograms are shown (left panels). The stimulation index was calculated by dividing the gMFI from stimulated conditions by the gMFI under unstimulated conditions (right panel). The horizontal dashed gray line indicates the value 1. N = 14 (pSYK), N = 15 (pPI3K p85), and N = 13 (pERK1/2). The horizontal line in the boxplot represents the median with jitter points for individual data points. Differences between unstimulated and stimulated samples were analyzed by an unpaired *t*-test or a Mann-Whitney *U*. When comparing more than two groups, a one-way ANOVA with Tukey's multiple comparisons test or a Kruskal-Wallis with Dunn's test to correct for multiple comparisons was used. ∗∗*P* < 0.01, ∗∗∗*P* < 0.001; ns: not significant.

The expansion of DN2 B cells in peripheral blood of r-axSpA patients prompted us to investigate their BCR signaling responsiveness. Because CD21^lo^ B cells (which include the DN2 and DN3 B cell fractions) are associated with anergy [14,15], we first studied BCR signaling responses among DN1, DN2, and DN3 B cell subsets in HCs. To this end, PBMCs were incubated *in vitro* with α-Ig or were left unstimulated. Subsequently, the phosphorylation levels of proximal BCR signaling proteins SYK and PI3K, as well as ERK1/2, a protein involved more distally from the BCR in the mitogen-activated protein kinase (MAPK) signaling pathway, were determined in the different B cell subsets using phosphoflow cytometry. Under unstimulated conditions, SYK and PI3K phosphorylation was significantly higher in DN2 B cells compared with DN1 and DN3 B cells (Fig. 3). Basal ERK1/2 phosphorylation was higher in both DN2 and DN3 B cells compared with DN1 B cells. BCR stimulation induced robust phosphorylation of SYK, PI3K, and ERK1/2 in DN1 B cells. This was reflected by a median stimulation index (SI) of 5.0 (pSYK), 3.4 (pPI3K), and 4.3 (pERK1/2) (see Fig. 3). On the contrary, BCR stimulation did not result in upregulation of SYK and PI3K phosphorylation in DN2 and DN3 B cells, as demonstrated by a median SI of around 1 for both subsets (Fig. 3). ERK1/2 phosphorylation was enhanced following BCR stimulation in DN2 and DN3 B cells, though responses were significantly lower than those in DN1 B cells, as exemplified by a median stimulation index of 1.6 (DN2) and 2.0 (DN3) (see Fig. 3).

Together, we observed that DN2 and DN3 B cells, but not DN1 cells, have an anergic profile in HCs.

### 3.4 DN2 B cells from r-axSpA patients display aberrant BCR signaling responses

**Fig. 4:**
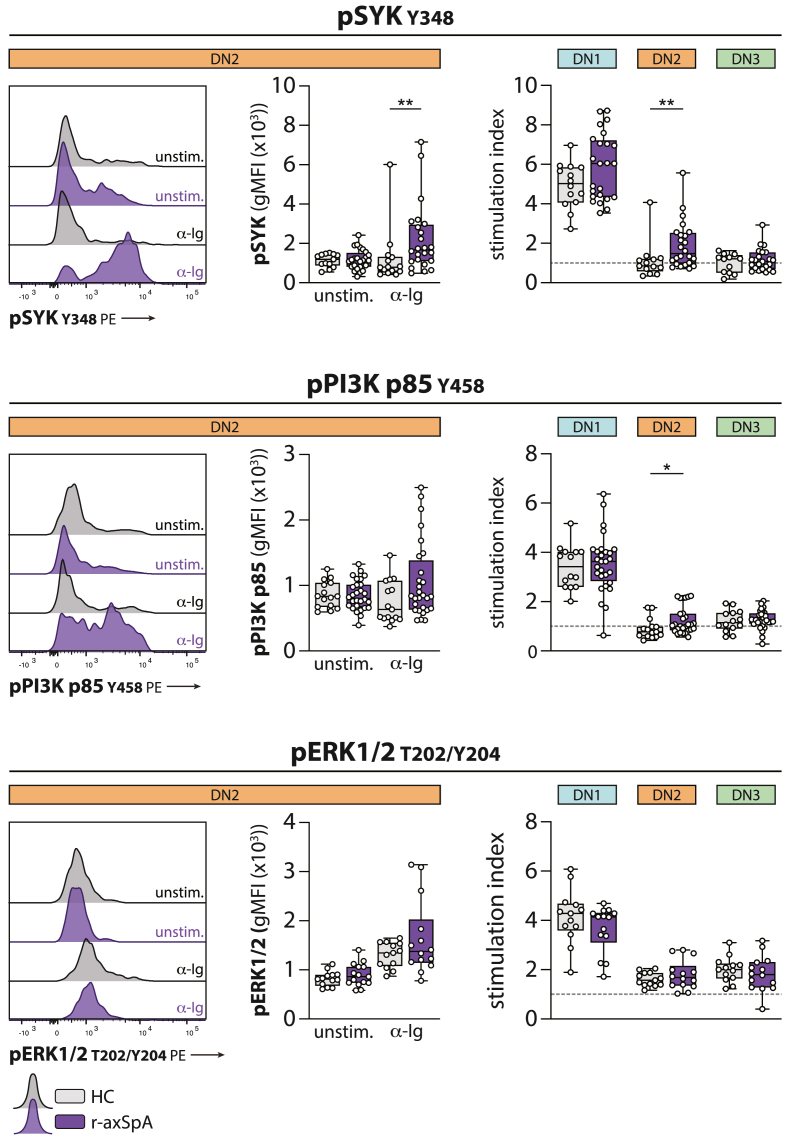
**Reinvigorated BCR signaling responses in DN2 B cells from r-axSpA patients.** The phosphorylation of SYK (Y348), PI3K p85 (Y458), and ERK1/2 (T202/Y204) was measured under unstimulated conditions (unstim.) and following BCR stimulation (α-Ig). Representative histograms are shown (left panels). The stimulation index was calculated by dividing the gMFI from stimulated conditions by the gMFI under unstimulated conditions (right panel). N = 14 and N = 24 (pSYK), N = 15 and N = 28 (pPI3K p85), and N = 13 and N = 15 (pERK1/2) for HCs and r-axSpA patients, respectively. The horizontal line in the boxplots represent the median with jitter points for individual data points. Differences were analyzed by an unpaired *t*-test or Mann-Whitney *U*. ∗*P* < 0.05; ∗∗*P* < 0.01.

We compared the observed BCR signaling responses in the DN B cell subsets form HCs (see Fig. 3) with those from r-axSpA patients. At unstimulated conditions, the phosphorylation level of SYK and ERK1/2 in all three DN B cell subsets showed no differences with HCs. A small but significant decrease in PI3K phosphorylation in DN1 and DN3 B cells from r-axSpA patients was found at rest (Suppl. Fig. 3). Interestingly, upon BCR stimulation, DN2 B cells showed a significant BCR signaling response for SYK and PI3K in r-axSpA patients, whereas this was absent in HCs (Fig. 4). Importantly, these alterations were specific for the DN2 B cell population, as DN1 BCR signaling responses were similar between r-axSpA patients and controls, and the anergic profile in DN3 B cells was unaltered in r-axSpA patients (Suppl. Fig. 3).

Taken together, DN2 B cells from r-axSpA patients displayed significant proximal BCR signaling responses, in contrast to DN2 B cells from HCs.

### 3.5 Enhanced BCR signaling responses in naïve and transitional B cells from r-axSpA patients

**Fig. 5:**
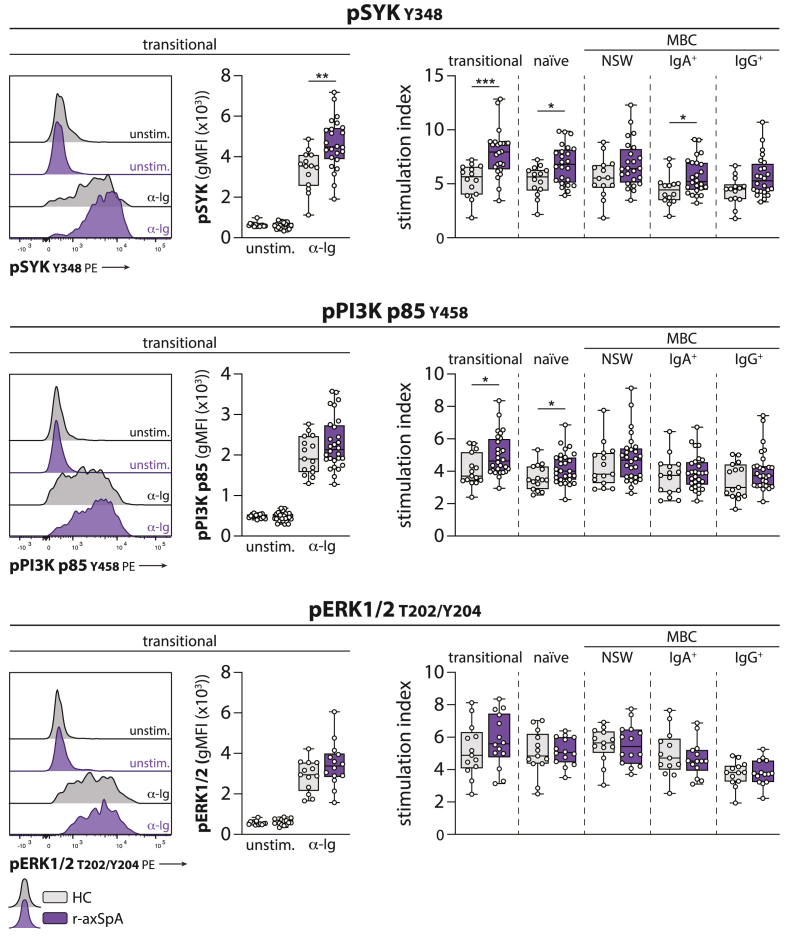
**Enhanced proximal BCR signaling responses in naïve and transitional B cells from r-axSpA patients.** The phosphorylation of SYK (Y348), PI3K p85 (Y458), and ERK1/2 (T202/Y204) was measured under unstimulated conditions (unstim.) and following BCR stimulation (α-Ig). Results are shown for transitional B cells with representative histograms (left panel). The stimulation index was calculated by dividing the gMFI from stimulated conditions by the gMFI under unstimulated conditions (right panel). N = 14 and N = 24 (pSYK), N = 15 and N = 28 (pPI3K p85), and N = 13 and N = 15 (pERK1/2) for HCs and r-axSpA patients, respectively. The horizontal line in the boxplots represent the median with jitter points for individual data points. Differences were analyzed by an unpaired *t*-test or Mann-Whitney *U*. ∗*P* < 0.05; ∗∗*P* < 0.01; ∗∗∗*P* < 0.001.

In the context of autoimmunity, extrafollicular-derived activated naïve B cells have been suggested as a possible source of cells differentiating into pathogenic DN2 B cells [13]. In addition, enhanced BCR signaling in antigen-naïve B cells is a common finding in systemic autoimmune conditions, such as GPA and RA [18,20,29]. Therefore, we hypothesized that the aberrant BCR signaling responses in the DN2 lineage could be the result of enhanced responses in the antigen-naïve B cell population in r-axSpA patients. The stimulation index of pSYK and pPI3K was indeed significantly increased in both transitional and naïve B cell populations from r-axSpA patients compared with HCs, indicating an enhanced BCR signaling responsiveness (Fig. 5).

The expansion of an activated naïve B cell population, characterized by CD21^lo^CD11c^+^ expression, has recently been described in SLE patients [30]. These cells were shown to express an autoreactive BCR and share a differentiational link with DN2 B cells [13,30]. Interestingly, in our cohort, CD21^lo^CD11c^+^ B cells within the transitional-naïve (CD27^−^IgM^+^) B cell population from r-axSpA patients also demonstrated an enhanced BCR signaling responsiveness (see Suppl. Fig. 4). In various MBC subpopulations, the BCR stimulation-induced SYK and PI3K phosphorylation tended to be slightly higher in r-axSpA than in HCs, which reached significance for SYK in IgA + MBCs (Fig. 5). The responsiveness of ERK1/2 was in none of the subsets significantly altered in r-axSpA patients.

Together, antigen-inexperienced transitional and naïve B cells from r-axSpA patients exhibited significantly enhanced BCR signaling responses, paralleling previous findings in autoimmune conditions.

### 3.6 BCR signaling responses in DN2 B cells correlate with r-axSpA disease activity parameters

**Fig. 6:**
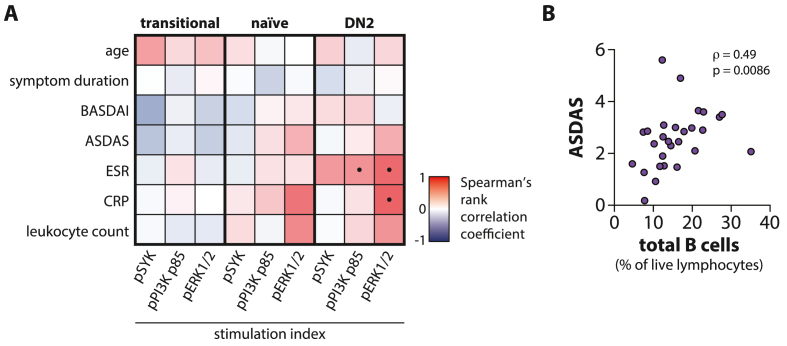
**Spearman's rank correlation analysis of BCR signaling responses with r-axSpA clinical parameters.** (**A**) Spearman's rank correlation coefficient matrix of the stimulation indexes for transitional, naïve, and DN2 B cells from r-axSpA patients with clinical features. These comprise age, symptom duration, Bath Ankylosing Spondylitis Disease Activity Index (BASDAI), Ankylosing Spondylitis Disease Activity Score (ASDAS), erythrocyte sedimentation rate (ESR), C-reactive protein (CRP), and leukocyte count. Dots indicate significant correlations (P < 0.05) (**B**) Spearman's rank correlation analysis of the frequency of circulating B cells with the ASDAS in r-axSpA patients. N = 24 (pSYK), N = 28 (pPI3K p85), and N = 15 (pERK1/2).

We explored whether the enhanced BCR responses in transitional, naïve, and DN2 B cells from r-axSpA patients statistically correlated with disease activity. Therefore, stimulation indexes of pSYK, pPI3K, and pERK1/2 from these three B cell populations were correlated with relevant clinical parameters. For transitional B cells, the pSYK stimulation index showed a moderately negative correlation (*ρ* = −0.46) with the Bath Ankylosing Spondylitis Disease Activity Index (BASDAI). For DN2 B cells, pSYK, pPI3K, and pERK1/2 stimulation indices were positively associated with the erythrocyte sedimentation rate (ESR; *ρ* = 0.34, 0.39, 0.60, respectively), as depicted in Fig. 6A. When r-axSpA patients were categorized (yes/no) based on having a history of ESM, Ankylosing Spondylitis Disease Activity Score (ASDAS) ≤2.1, or CRP levels ≤5 mg/L we did not observe differences in BCR signaling responses (data not shown).

Finally, we explored possible correlations between circulating B cell subset frequencies and clinical parameters. In r-axSpA patients, the proportions of total B cells correlated positively with the ASDAS (*ρ* = 0.49) (Fig. 6B). A negative correlation of proportions of total B cells with age was found in r-axSpA patients, but not in HCs (*ρ* = −0.47 and *ρ* = −0.03, respectively; Suppl. Fig 5). Although weak in strength, total B cell frequencies, ESR, CRP, and leukocyte count inversely associated with both age and symptom duration (Suppl. Fig 5). While no other evident associations were observed between any of the subsets within the B cell compartment and clinical parameters, there was evidence for strong correlations amongst subset frequencies. DN2 B cell frequencies strongly correlated with class-switched MBC and PB frequencies in r-axSpA patients (*ρ* = 0.70 and *ρ* = 0.62; see Suppl Fig 5.). In HCs, similar findings were observed, although lower in strength.

Altogether, these results demonstrate statistical correlations between aberrant BCR signaling responses in r-axSpA patients and clinical disease activity parameters, ESR in particular. Also, r-axSpA patients that are relatively young and more recently diagnosed appear to be characterized by higher disease activity, as reflected by the ASDAS, which is accompanied by elevated frequencies of circulating B cells.

## 4 Discussion

In this study we analyzed the composition of the peripheral B cell compartment and BCR signaling responses in distinct B cell subpopulations in both HCs and patients with r-axSpA. We observed an expansion of circulating DN2 B cells in r-axSpA patients compared with HCs. These DN2 B cells from r-axSpA patients also demonstrated with an enhanced BCR signaling responsiveness, in contrast to HCs, were these cells remained unresponsive. Also, transitional and naïve B cells from r-axSpA patients showed elevated BCR signaling responses compared with HCs. To our knowledge, this is the first study describing aberrant BCR signaling responses in DN2 B cells in r-axSpA patients.

Previous studies have used the presence or absence of different markers, such as IgD, CD27, CXCR5, T-bet, and FCRL5 - or a combination of these markers - to study CD21^lo^ B cells. Depending on their exact phenotype, these cells have been termed, among others, atypical memory, age-associated, or DN2 and DN3 B cells [8,9,31]. Although both DN2 and DN3 B cell populations lack or express low levels of CD21, DN2 B cells expresses additional activation markers such as CD11c, T-bet, and FcRL5, that distinguish these cells phenotypically and possibly functionally from DN3 B cells [13,32]. Regarding BCR signaling, CD21^lo^ B cells have been shown to exhibit elevated BCR signalosome phosphorylation at rest, attributed to increased BCR signalosome protein expression [33]. However, their response towards BCR and TLR9 stimuli is impaired in healthy individuals [13,16], consistent with the anergic phenotype observed in our DN2 and DN3 B cell populations. In contrast, BCR signaling responses in DN2 B cells, but not DN3 B cells, from r-axSpA patients were enhanced. These findings indicate that, unlike DN3 B cells, DN2 B cells demonstrate a reinvigorated BCR signaling response in r-axSpA compared with HCs.

BCR signaling is known to be a tightly regulated process. In anergic B cells, due to chronic BCR stimulation, SYK phosphorylation and PI3K function is actively repressed by regulatory phosphatases, such as SHP-1, SHIP-1, PTEN, and PTPN22 [34]. Increased expression of these phosphatases is an important driver of dampening BCR signaling responses. Also, inhibitory receptors, such as the FcγRIIb, play an important role in facilitating anergy by activating these phosphatases and by blocking interactions between the BCR and co-receptors such as CD19 [35]. In future studies, alterations in the expression and/or activity of these phosphatases should be investigated in r-axSpA, as such alterations have been associated with enhanced BCR signaling activity in autoimmune disease [29,36].

The increased frequencies of circulating DN2 B cells observed in our r-axSpA cohort are also reported in systemic autoimmune diseases [13,37], such as RA and SLE, and validate our previous observation of increased CD27^–^CD21^lo^ B cell frequencies in another r-axSpA study [7]. In terms of BCR signaling responses, r-axSpA patients show similarities with SLE patients, where hyperactivated PI3K/Akt/mTOR signaling has been described in CD24^-^CD20^hi^ B cells characterized by a CD27^-^CD21^lo^CD11c^+^T-bet^+^ phenotype [38]. Because CD19 and CD20 are highly expressed by DN2 B cells, the observed increase in *MS4A1* expression in total B cells from r-axSpA patients could reflect an enlarged DN2 population [39]. The enhanced *MTOR* expression in B cells from r-axSpA patients further strengthens our functional findings of hyperactivated PI3K signaling in r-axSpA B cells.

In autoimmune diseases such as SLE, activated naïve B cells possibly escape peripheral tolerance checkpoints and differentiate into DN2 B cells, which are poised towards ASC differentiation [13,40]. Both T-dependent and T-independent factors (e.g., CD40L, IL-21, IFN-γ, and TLR7 stimulation) as well as BCR signals are crucial drivers of DN2 B cell differentiation [13,41,42]. Elevated IL-21 and IFN-γ serum levels have been observed in r-axSpA patients [43,44]. In addition to presence of these soluble factors, enhanced activity of the PI3K/Akt/mTOR pathway may guide B cell fate decision towards extrafollicular responses and subsequent DN2 B cell differentiation instead of entering the germinal center [45,46]. Interestingly, aberrant BCR signaling responses were also present in transitional, naïve, and particularly also in the CD21^lo^CD11c^+^ transitional-naïve B cell population in r-axSpA patients. A study by Jenks et al. demonstrated that activated naïve B cells, characterized by a CD21^lo^CD11c^+^ phenotype, share phenotypical and functional features with DN2 B cells [13]. Moreover, in this study, the activated naïve B cells showed clonal overlap with both DN2 B cells and PBs [13]. Our findings suggest that altered signaling properties in transitional-naïve CD27^−^IgM^+^ B cells, perhaps because of a chronic inflammatory environment in patients with immune-mediated diseases, may contribute to enhanced activation and potentially differentiation towards DN2 B cells. Because the DN2 B cell population may harbor pathogenic ASC-precursor cells in autoimmune diseases [13,37,47], these cells may be a potential source of autoantibody secreting cells in r-axSpA pathogenesis. DN2 B cells may also contribute to the pathogenesis through antigen presentation and T cell polarization, like in RA [48].

Previous studies have reported elevated total IgA and autoreactive IgA in serum of r-axSpA patients [[49], [50], [51], [52]]. We therefore speculate that an increased frequency of circulating DN2 B cells with aberrant BCR signaling responses in r-axSpA patients might form a source of (possibly autoreactive) ASC-precursors. Because r-axSpA patients show increased IgA switched cells among DN2 B cell fraction and increased BCR-induced SYK phosphorylation in IgA^+^ B cells, it is conceivable that IgA^+^ DN2 cells develop through dysregulated mucosal immune responses. These immune responses potentially originate from gut inflammation in r-axSpA, as roughly half of the patients shows subclinical gut inflammation and patients are at a higher risk of developing inflammatory bowel disease [[53], [54], [55]].

Our study has some limitations. First, our study is explorative, and the cohort size used for RNA sequencing of B cells from r-axSpA patients and HCs was relatively small, hampering our ability to compare clinical phenotypes within the r-axSpA patient group. Secondly, as bulk RNA sequencing was performed on total B cells, our analysis could not specifically address the contributions of individual subsets such as DN2 B cells, which constitute only a minor fraction of the total B cell population. Thirdly, the limited availability of cells restricted the number of signaling proteins that we could include in our BCR signaling analyses, offering only a partial view of the complexities inherent to the downstream BCR signaling pathways. Nonetheless, the measurement of SYK and PI3K, two crucial kinases guiding BCR signaling, allowed us to gain insight into proximal BCR signaling responses. However, caution should be taken when interpreting findings related to more downstream BCR signaling, as a limited number of patients were included for the analysis of ERK1/2, and other downstream routes, such as the NF-κB pathway, were not investigated. Also, because of relatively low counts for individual DN B cell subsets, our findings on altered BCR signaling responses in these subsets should be interpreted with care. Lastly, data were obtained from cryopreserved PBMCs. As some B cell subpopulations (e.g., ASCs) are more sensitive to freezing and thawing than others, this may have affected the observed B cell frequencies.

In conclusion, our study indicates that DN2 and DN3 B cells are anergic in healthy individuals. In r-axSpA, however, DN2 B cells - but not DN3 B cells – are expanded and significantly respond to BCR stimulation, which may predispose these cells for more efficient differentiation into ASC. Because transitional and naïve B cells from r-axSpA patients also exhibited heightened sensitivity to BCR stimulation, our results are suggestive of a potential differentiative relationship among these subsets. Altogether, because of the similarities in aberrant BCR signaling responses between systemic autoimmune disease and r-axSpA patients [20,29,56], our data support the involvement of B cells in r-axSpA pathogenesis. Further research is needed to investigate mechanistic links between the observed B cell abnormalities and the pathogenesis of r-axSpA.

## CRediT authorship contribution statement

**Rick Wilbrink:** Writing – review & editing, Writing – original draft, Visualization, Validation, Software, Project administration, Methodology, Investigation, Funding acquisition, Formal analysis, Data curation, Conceptualization. **Stefan F.H. Neys:** Writing – review & editing, Writing – original draft, Visualization, Validation, Software, Project administration, Methodology, Investigation, Formal analysis, Data curation, Conceptualization. **Rudi W. Hendriks:** Writing – review & editing, Supervision, Resources, Funding acquisition, Conceptualization. **Anneke Spoorenberg:** Writing – review & editing, Supervision, Methodology, Conceptualization. **Frans G.M. Kroese:** Writing – review & editing, Supervision, Funding acquisition, Conceptualization. **Odilia B.J. Corneth:** Writing – review & editing, Supervision, Methodology, Funding acquisition, Conceptualization. **Gwenny M.P.J. Verstappen:** Writing – review & editing, Supervision, Methodology, Funding acquisition, Conceptualization.

## Funding

This research was funded by the Target2B consortium and an internal grant from the Erasmus MC (MRace), and is part of the Veni research program of 10.13039/100008117Gwenny M.P.J. Verstappen [project number 09150162010166], which is financed by the 10.13039/501100003246Dutch Research Council (10.13039/501100003246NWO).

## Declaration of competing interest

The authors declare that they have no known competing financial interests or personal relationships that could have appeared to influence the work reported in this paper.

## Data Availability

Data will be made available upon reasonable request.
