# Supplementary material for: Aberrant B cell receptor signaling responses in circulating double-negative 2 B cells from radiographic axial spondyloarthritis patients
Source: J Transl Autoimmun. 2025 Jan 23;10:100270. doi: 10.1016/j.jtauto.2025.100270 (PMC11835616; doi:10.1016/j.jtauto.2025.100270)
Supplement: Multimedia component 1 [file mmc1.docx]

**Supplementary Materials to:**

**Aberrant B Cell Receptor Signaling Responses in Circulating Double-Negative 2 B Cells from Radiographic Axial Spondyloarthritis Patients**

Rick Wilbrink^1,*^, Stefan F.H. Neys^2,*^, Rudi W. Hendriks^2^, Anneke Spoorenberg^1^, Frans G.M. Kroese^1^, Odilia B.J. Corneth^2,#^, Gwenny M.P.J. Verstappen^1,#^

^1^Department of Rheumatology and Clinical Immunology, University of Groningen, University Medical Center Groningen, Groningen, The Netherlands

^2^Department of Pulmonary Medicine, Erasmus MC, University Medical Center, Rotterdam, The Netherlands

*shared first author.

^#^shared last author.

**Supplementary Table 1.** Patient and healthy control characteristics of the two independent experimental cohorts

| **Characteristics** | **Experimental cohort 1: RNA-bulk sequencing** | | **Experimental cohort 2: phosphoflow cytometry** | |
| --- | --- | --- | --- | --- |
| Participant status | r-axSpA | HC | r-axSpA | HC |
| Number of participants (n) | 8 | 8 | 28 | 15 |
| Age (years) (mean ± SD) | 48 ± 10 | 48 ± 21 | 50 ± 14 | 50 ± 16 |
| Male sex assigned at birth (n, %) | 4 (50) | 5 (63) | 21 (75) | 11 (73) |
| Symptom duration (years) (median, Q1-Q3) | 24 (14 – 27) |  | 24 (8 – 31) |  |
| HLA-B27 positive (n, %) | 8 (100) |  | 25 (89) |  |
| ASDAS (mean ± SD) | 2.5 ± 0.8 |  | 2.6 ± 1.1 |  |
| ASDAS > 2.1 (n, %) | 7 (88) |  | 17 (61) |  |
| BASDAI (mean ± SD) | 5.1 ± 2.7 |  | 3.9 ± 2.2 |  |
| ESR (mm/hr) (median, Q1-Q3) | 15.0 (11.5 – 26.5) |  | 17.5 (10.5 – 42.0) |  |
| CRP (mg/L) (median, Q1-Q3) | 1.9 (0.8 – 5.5) |  | 6.0 (2.2 – 11.0) |  |
| Leukocytes (10.0^9^/L) (median, Q1-Q3) | 7.9 (6.5 – 9.3) |  | 6.7 (5.3 – 8.5) |  |
| Current biologic, targeted synthetic and conventional DMARD use* (n, %) | 0 (0) |  | 0 (0) |  |
| History of biologic DMARD use (n, %) | 4 (50) |  | 8 (29) |  |
| History of ESM (n, %): | 2 (25) |  | 14 (50) |  |
| Uveitis (n, %) | 2 (25) |  | 10 (36) |  |
| Inflammatory bowel disease (n, %) | 1 (10) |  | 2 (7) |  |
| Psoriasis (n, %) | 0 (0) |  | 2 (7) |  |
| History of PM (n, %): | 2 (25) |  | 17 (61) |  |
| Arthritis (n, %) | 0 (0) |  | 11 (3) |  |
| Dactylitis (n, %) | 1 (13) |  | 5 (18) |  |
| Enthesitis (n, %) | 1 (13) |  | 6 (21) |  |

Data are presented as number of patients (%), mean ± SD or median (Q1 – Q3); r-axSpA = Radiographic axial spondyloarthritis; HC = Healthy controls; DMARD = Disease modifying antirheumatic drugs; HLA-B27 = Human leukocyte antigen B27; ASDAS = Ankylosing Spondylitis Disease Activity Score; BASDAI = Bath Ankylosing Spondylitis Disease Activity Index; ESM = extra skeletal-manifestations; PM = peripheral manifestations. *biologic (e.g., anti-TNFα), synthetic (e.g., JAK/STAT-inhibitors) and conventional DMARD use.

| **Marker or target** | **Label** | **Clone** | **Company** |
| --- | --- | --- | --- |
| *Antibodies used for B cell sorting* | | | |
| CD19 | FITC | HIB19 | Biolegend |
| CD20 | PE | 2H7 | eBioscience |
| *Antibodies used for the detection of different B cells subsets* | | | |
| Human Trustain FcX | - | - | Biolegend |
| CD11c | BV480 | B-LY6 | BD Biosciences |
| CD19 | BV750 | HIB19 | BD Biosciences |
| CD21 | Biotin | Bu32 | Biolegend |
| CD3 | AF700 | UCHT1 | Invitrogen |
| CD27 | BV421 | L128 | BD Biosciences |
| CD38 | BV786 | HB7 | BD Biosciences |
| IgA | FITC | Polyclonal | Southern Biotech |
| IgD | BV711 | IA6-2 | BD Biosciences |
| IgM | PerCP-Cy5.5 | G20-127 | BD Biosciences |
| IgG | PE-Cy7 | G18-145 | BD Biosciences |
| Streptavidin | APC | - | Biolegend |
| *Antibodies used for the quantification of phosphorylated protein* | | | |
| pPI3K p85 | - | E3UI1H | Cell Signaling Technologies |
| pSYK | PE | I120-722 | BD Biosciences |
| pERK1/2 | PE | 20A | BD Biosciences |
| Donkey-anti-Rabbit | PE | - | Jackson Immunolaboratories |

**Supplementary Table 2.** Antibodies utilized for B cell sorting and phosphoflow cytometry analysis

| **Pathway** | **Genes** |
| --- | --- |
| B cell Receptor Signaling | AKT1, AKT2, AKT3, BCL10, BLK, BLNK, BTK, CARD11, CBL, CD19, CD22, CD79A, CD81, CHUK, CR2, CUL1, FBXW11, FCGR2B, FCRL1, FCRL3, FCRL4, FCRL5, GRB2, GSK3B, HRAS, IFITM1, IKBKB, IKBKG, ITPR3, JUN, KRAS, LYN, MALT1, MAP2K1, MAP3K7, MAPK3, NFATC1, NFATC2, NFKB1, NFKBIA, NFKBIB, NFKBIE, NRAS, ORAI1, PIK3CA, PIK3CD, PIK3R2, PLCG1, PLCG2, PSMB10, PSMB8, PSMB9, PTPN18, PTPN6, RAC1, RELA, SKP1, SYK, UBA52, VAV1 |
| Fc Receptors and Phagocytosis | BCL10, BTK, CARD11, CD163, CD164, CHUK, CUL1, FCAR, FCGR1A, FCGR2B, FCGR3A/B, FCN1, FCRL1, FCRL2, FCRL3, FCRL4, FCRL5, GRB2, HRAS, IKBKB, IKBKG, ITK, ITPR3, JUN, KRAS, LAT, LYN, MALT1, MAP3K7, MAPK3, MS4A2, NFATC1, NFATC2, NFKB1, NFKBIA, NRAS, PIK3CA, PIK3R2, PLCG1, PLCG2, PRKCQ, PSMB10, PSMB8, PSMB9, RAC1, RELA, SKP1, SYK, TAB1, TAB2, TAB3, TRAF6, UBA52, UBB, VAV1 |
| Autoantigens | ENO1, GRN, H2AFY2, HIST1H2AC, HIST1H2AE, HIST1H2BD, HIST1H2BF, HIST1H2BK, HIST1H3G, HIST1H3H, HIST1H4E, HIST1H4H, IMPDH2, MBP, PARP1, POLR1C, POLR1D, POLR2A, POLR3D, POLR3H, SP100, TG, TROVE2 |

**Supplementary Table 3.** Genes within pathways significantly elevated in radiographic axial spondyloarthritis patients compared with healthy controls.

The genes and pathways are part of the nCounter® Human AutoImmune Profiling Panel

**
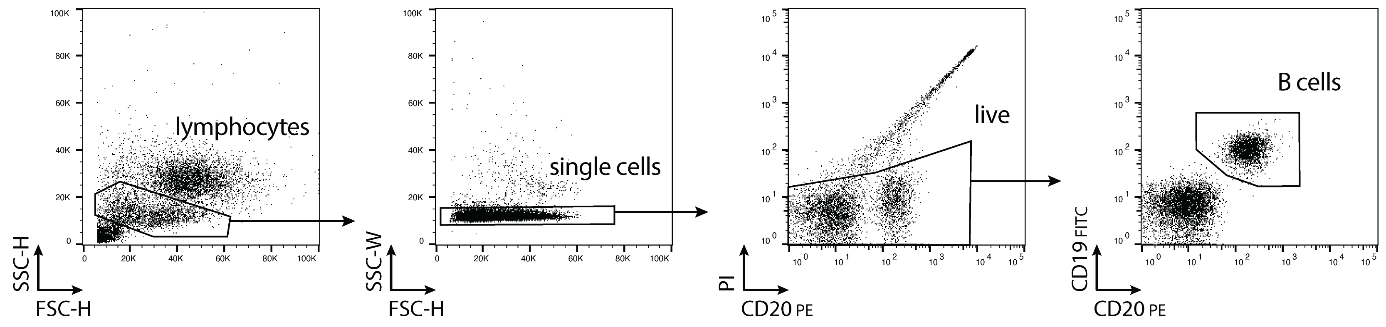
**

**Supplementary Figure 1.** The applied gating strategy for sorting of CD19^+^CD20^+^ B cells. PI, propidium iodine.


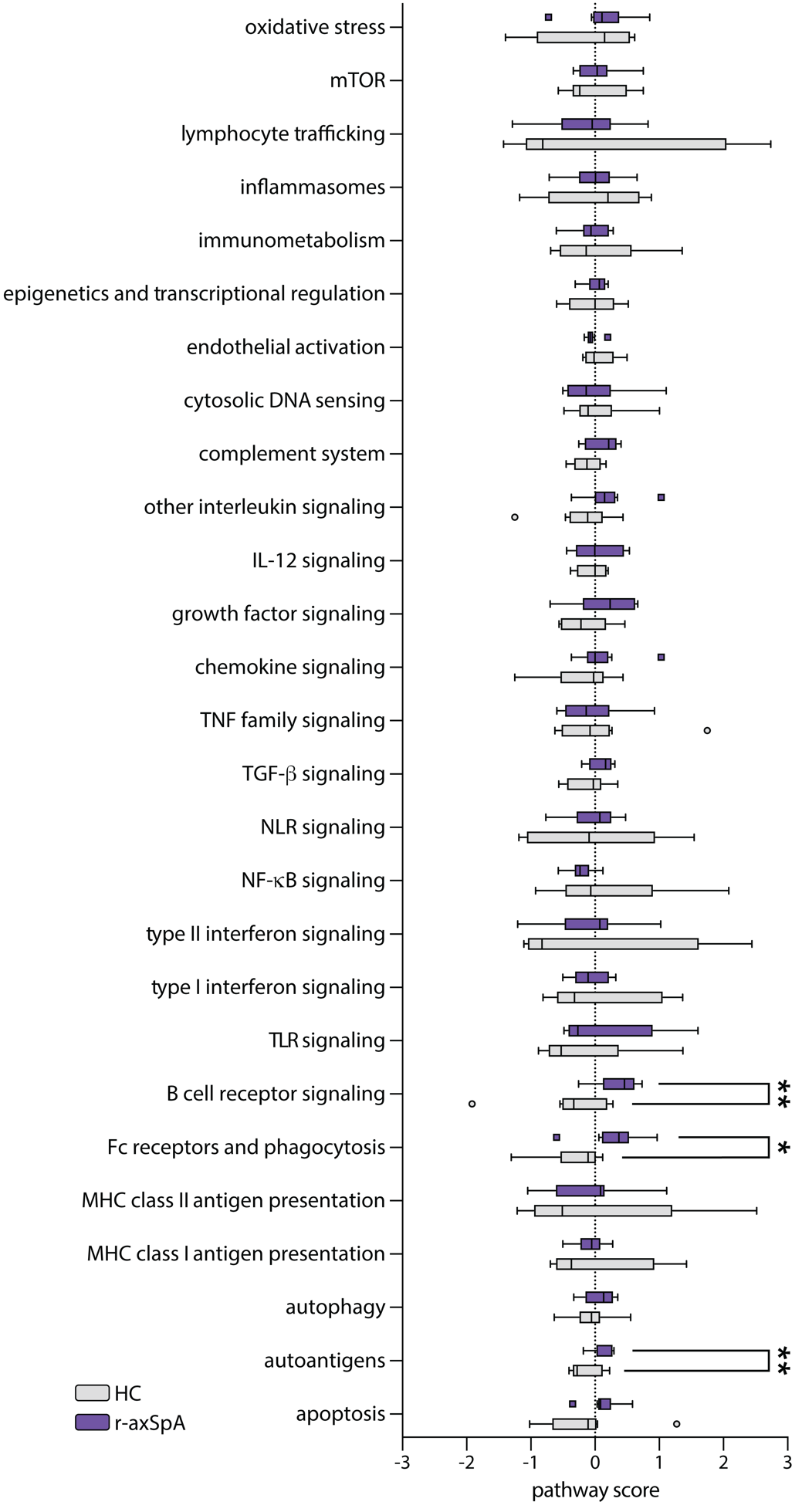


**Supplementary Figure 2.** Pathway scores displaying multiple biological pathways, as reported in the Human Autoimmune Profiling Panel generated by Nanostring Technologies. The horizontal line in the boxplot represents the median and points displaying values outside the whiskers. N=8 for both HC and r-axSpA patients. Differences between HCs and r-axSpA patients were analyzed by a Mann-Whitney *U*. **P*<0.05; ***P*<0.01


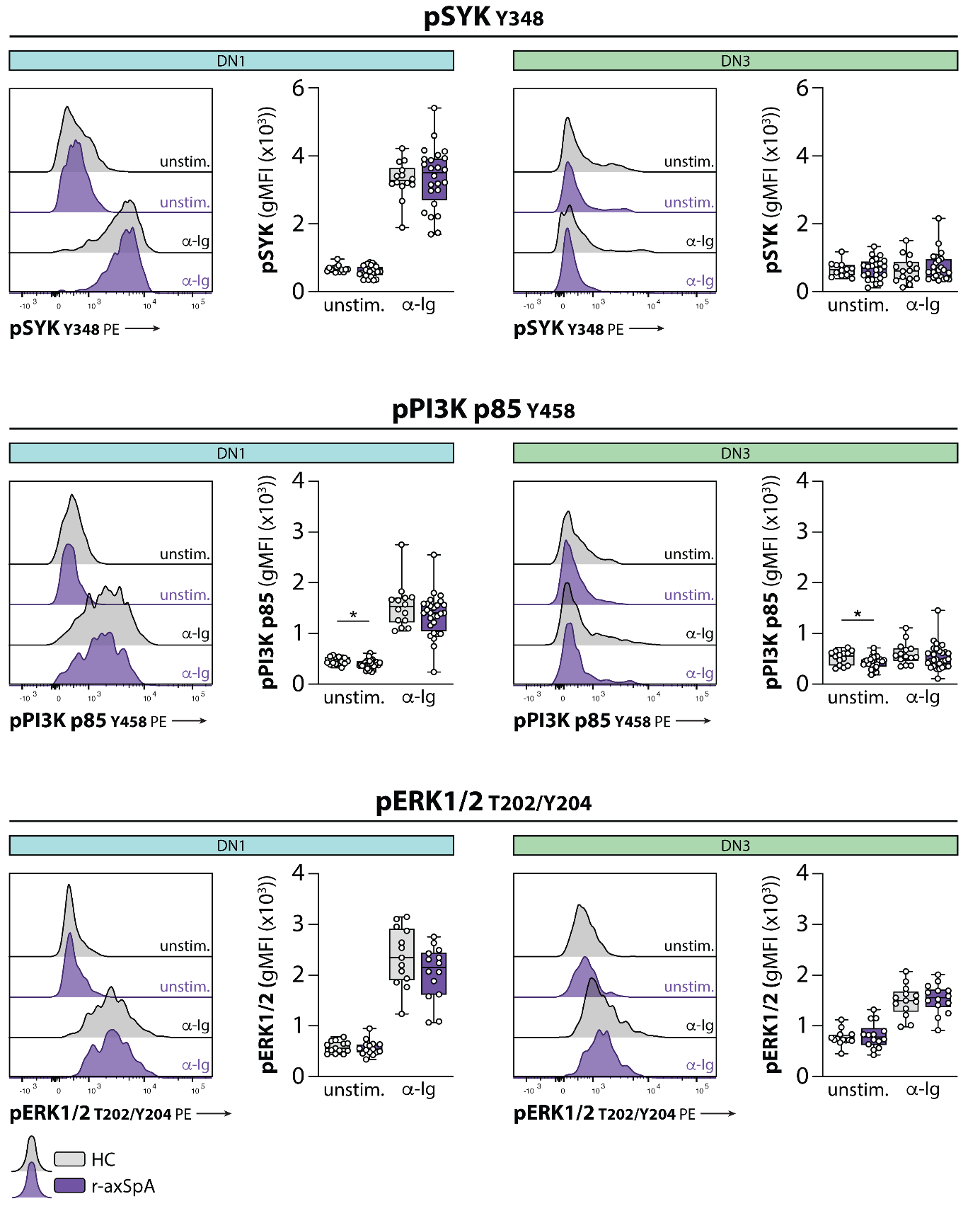


**Supplementary Figure 3.** The phosphorylation of SYK (Y348), PI3K p85 (Y458), and ERK1/2 (T202/Y204) was measured under unstimulated conditions (unstim.) and following BCR stimulation (α-Ig). Representative histograms are shown on the left. N=14 and N=24 (pSYK), N=15 and N=28 (pPI3K p85), and N=13 and N=15 (pERK1/2) for HCs and r-axSpA patients, respectively. The horizontal line in the boxplots represent the median with jitter points for individual data points. Differences were analyzed by an unpaired *t*-test. **P*<0.05.

**
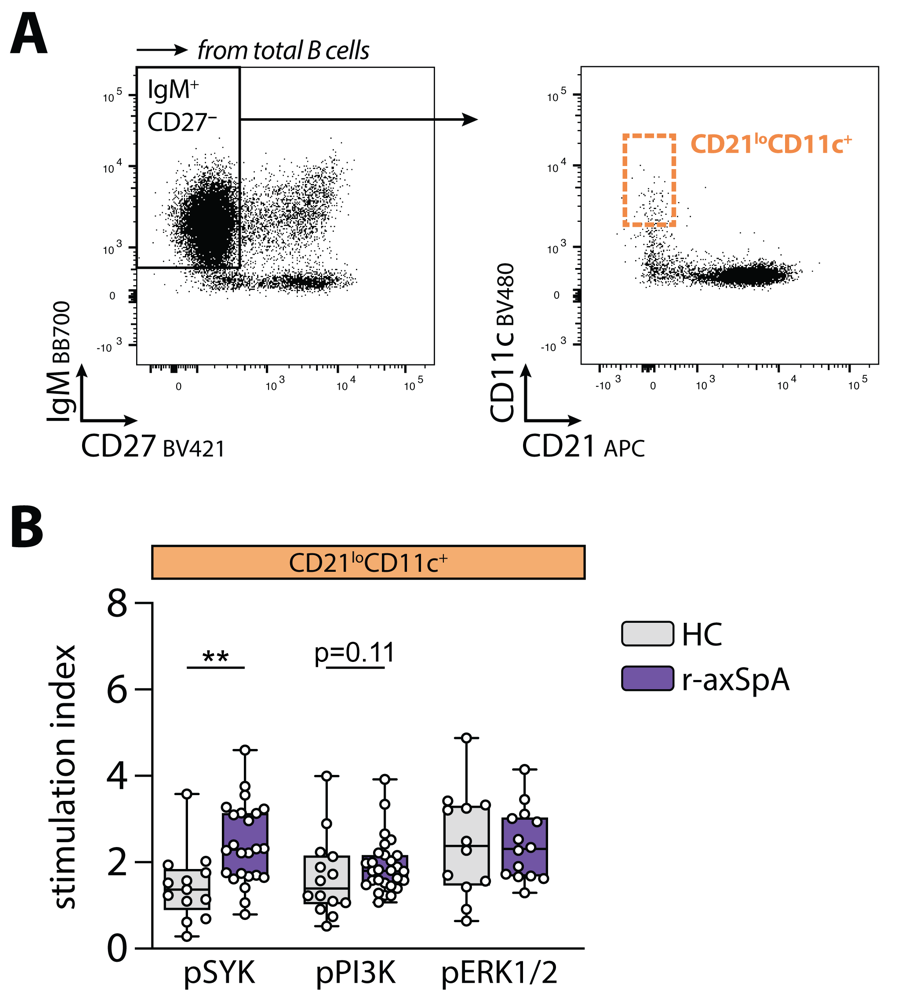
**

**Supplementary Figure 4.** BCR signaling responses in CD21^lo^CD11c^+^ transitional and naïve B cells. (**A**) Gating strategy for the detection of IgM^+^CD27^–^CD21^lo^CD11c^+^ transitional and naïve B cells. (**B**) The stimulation index of SYK (Y348), PI3K p85 (Y458), and ERK1/2 (T202/Y204) was calculated by dividing the gMFI from stimulated conditions by the gMFI under unstimulated conditions. N=14 and N=24 (pSYK), N=15 and N=28 (pPI3K p85), and N=13 and N=15 (pERK1/2) for HCs and r-axSpA patients, respectively. The horizontal line in the boxplots represent the median with jitter points for individual data points. Differences were analyzed by an unpaired *t*-test or Mann-Whitney *U*. **P*<0.05; ***P*<0.01.


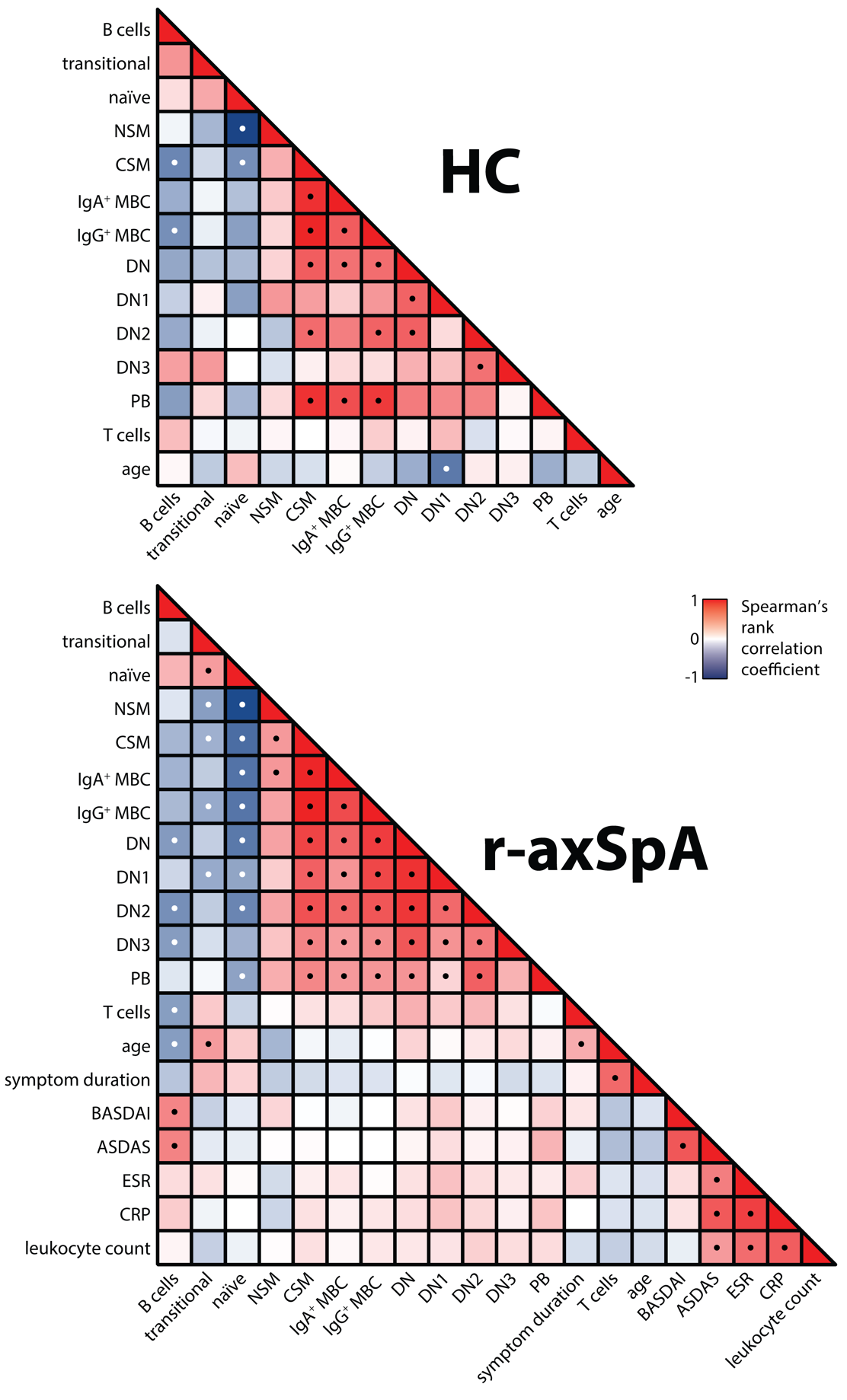


**Supplementary Figure 5.** Spearman’s rank correlation coefficient matrices for HC and r-axSpA patients of multiple B cells subsets and clinical parameters. r-axSpA clinical parameters comprise age, symptom duration*,* Bath Ankylosing Spondylitis Disease Activity Index (BASDAI), Ankylosing Spondylitis Disease Activity Score (ASDAS), erythrocyte sedimentation rate (ESR), C-reactive protein (CRP), and leukocyte count. Dots indicate significant correlations (P<0.05).
